# *In Vitro* activity of novel glycopolymer against clinical isolates of multidrug-resistant *Staphylococcus aureus*

**DOI:** 10.1371/journal.pone.0191522

**Published:** 2018-01-17

**Authors:** Vidya P. Narayanaswamy, Scott A. Giatpaiboon, John Uhrig, Paul Orwin, William Wiesmann, Shenda M. Baker, Stacy M. Townsend

**Affiliations:** 1 Synedgen, Inc., Claremont, California, United States of America; 2 Medical College of Wisconsin, Wisconsin, United States of America; 3 California State University, San Bernardino, United States of America; Institut National de la Recherche Agronomique, FRANCE

## Abstract

The incidence of multidrug-resistant (MDR) organisms, including methicillin-resistant *Staphylococcus aureus* (MRSA), is a serious threat to public health. Progress in developing new therapeutics is being outpaced by antibiotic resistance development, and alternative agents that rapidly permeabilize bacteria hold tremendous potential for treating MDR infections. A new class of glycopolymers includes polycationic poly-N (acetyl, arginyl) glucosamine (PAAG) is under development as an alternative to traditional antibiotic strategies to treat MRSA infections. This study demonstrates the antibacterial activity of PAAG against clinical isolates of methicillin and mupirocin-resistant *Staphylococcus aureus*. Multidrug-resistant *S*. *aureus* was rapidly killed by PAAG, which completely eradicated 88% (15/17) of all tested strains (6-log reduction in CFU) in ≤ 12-hours at doses that are non-toxic to mammalian cells. PAAG also sensitized all the clinical MRSA strains (17/17) to oxacillin as demonstrated by the observed reduction in the oxacillin MIC to below the antibiotic resistance breakpoint. The effect of PAAG and standard antibiotics including vancomycin, oxacillin, mupirocin and bacitracin on MRSA permeability was studied by measuring propidium iodide (PI) uptake by bacterial cells. Antimicrobial resistance studies showed that *S*. *aureus* developed resistance to PAAG at a rate slower than to mupirocin but similar to bacitracin. PAAG was observed to resensitize drug-resistant *S*. *aureus* strains sampled from passage 13 and 20 of the multi-passage resistance study, reducing MICs of mupirocin and bacitracin below their clinical sensitivity breakpoints. This class of bacterial permeabilizing glycopolymers may provide a new tool in the battle against multidrug-resistant bacteria.

## Introduction

*Staphylococcus aureus* is the most frequently isolated human bacterial pathogen associated with several severe clinical infections including soft-tissue and endovascular infections, pneumonia, and sepsis in patients frequenting hospitals or other healthcare facilities [1]. Over 2 million people every year in the US acquire serious infections with strains of *S*. *aureus* that are resistant to one or more of the antibiotics designed to treat those infections. At least 23,000 people in the US die each year as a direct result of these antibiotic-resistant infections. Misuse and overuse of antimicrobials has accelerated the prevalence of antimicrobial resistance and tolerance to the first-line drugs used to treat infections caused by *S*. *aureus*.

Methicillin resistant *S*. *aureus* (MRSA) is a well-known nosocomial pathogen often associated with health care-associated and community acquired infections [2] and is also a significant contributor to pulmonary decline in patients with cystic fibrosis [3, 4]. First described in 1960’s, MRSA continues to spread worldwide despite efforts of novel therapeutic development. MRSA isolates are resistant to available penicillin’s and other β-lactam antimicrobial drugs, limiting the potential treatment options with standard antibiotic therapy [5–8]. Patients affected by MRSA strains have an increased risk of poorer clinical outcomes, and 64% of them are more likely to die than patients infected with non-resistant strains of the same bacteria [9]. In 2013, MRSA was classified as a serious threat by the Centers for Disease Control and Prevention (CDC) [10]. Current research continues to provide evidence showing increasing incidence and virulence among resistant strains of *S*. *aureus*, despite the development of alternative strategies to combat MRSA infections [6, 11–14].

Standard of care treatments for multidrug-resistant *S*. *aureus* infections include vancomycin, linezolid, daptomycin and β-lactams. Vancomycin has been a mainstay of therapy for MRSA infections, but its effectiveness has been challenged by its extensive use, generating a selective pressure that favors the outgrowth of rare VRSA strains. Glycopeptides, like vancomycin and teicoplanin, have been effective bactericidal agents against MRSA infections but are often associated with adverse effects including renal failure and nephrotoxicity [15, 16]. The combination effect of vancomycin and β-lactam against *S*. *aureus* clinical isolates, including additive and/or synergistic effects [17–18] and antagonistic effects [19–22] have been contradictory.

Mupirocin, oxacillin, and bacitracin have been used as topical agents or intranasal treatments to prevent onset and spread of primary and secondary MRSA infections for over 10 years [23–27]. However, extensive use of mupirocin, bacitracin and oxacillin has also culminated in increased resistance in MRSA strains [28, 29], which is also consistently reported in community-associated MRSA infections [30]. Studies and clinical trials have also focused on antimicrobial peptides (AMP’s) for their wide-spectrum of antibacterial activity. However, many antimicrobial peptides are polycationic and have some cytotoxicity, limiting their use [31]. Recent studies suggest that a combination of vancomycin and ceftaroline possess greater bactericidal activity [21, 22], however, clinical experience with these therapies is limited. Ceftaroline is commonly associated with adverse effects such as skin rash, nausea, vomiting, and diarrhea neutropenia and agranulocytosis, particularly with high doses or prolonged exposure [32–35]. Development and successful implementation of novel, non-toxic strategies for treating emerging resistant pathogens is critical to limiting the potential increased lethality of *S*. *aureus*, minimizing the side effects of last-resort therapies, and reducing the spread of acquired nosocomial infections.

Poly-N (acetyl, arginyl) glucosamine (PAAG) represents a novel class of glycopolymers with antibacterial activity against a wide range of pathogenic Gram- negative and Gram- positive bacteria including MRSA, *Pseudomonas aeruginosa*, and non-tuberculous *Mycobacteria* (NTM) [36, 37]. Polyglucosamines are not commonly associated with toxicity [38–42]. PAAG has been shown to be versatile, surface-active glycopolymer with broad antimicrobial activity and excellent biocompatibility.

The goal of this study is to complete a preliminary assessment of the antibacterial efficacy of PAAG against clinical isolates of multidrug-resistant *S*. *aureus*, to evaluate PAAG’s capability to kill MRSA at its stationary phase of growth, to permeabilize bacteria, to re-sensitize MRSA isolates to conventional antibiotics and to study antimicrobial resistance development to PAAG, using the antibiotics mupirocin and bacitracin as comparators.

## Materials and methods

### Bacterial strains and culture conditions

Seventeen opportunistic pathogens of methicillin resistant *S*. *aureus* isolated from respiratory, skin and blood infections were used in the study. The MRSA strains designated SA01-SA12 were hospital acquired and obtained from California State University, San Bernardino (Paul Orwin). The mupirocin resistant MRSA strains (MMRSA) were obtained from Washington University, Minneapolis (Mike Dunn). *S*. *aureus* ATCC 6538 was used as a control in the microdilution test procedure for antibiotics. The experimental preparation of the cultures was adapted from CLSI [43]. Bacterial cultures were maintained in -80°C for up to 6 months. Luria-Bertani Broth (LB, BD) and Mueller Hinton broth (MHB, BD) media were used to grow the bacteria.

### Antimicrobial agents

Three antibiotics, oxacillin (TCI), bacitracin (Alfa Aesar) and mupirocin (USP) were used in the study. Stock solutions were freshly prepared from dry powders according to the manufacturer’s instructions. Synedgen's polycationic proprietary glycopolymer, PAAG, is an arginine derivative of a natural polysaccharide poly-N (acetyl, arginyl) glucosamine. It is polycationic and soluble at physiologic pH.

### *In vitro* susceptibility testing

Minimum inhibitory concentrations (MICs) of antibiotics and PAAG were determined with the broth microdilution technique described by the Clinical and Laboratory Standards Institute (CLSI). Each of the clinical isolates was tested against oxacillin (0.125–512 μg/mL), mupirocin (0.125–2048 μg/mL), and PAAG (8–2048 μg/mL). Serial two-fold dilutions of oxacillin, mupirocin, and PAAG were prepared in supplemented MH broth and 100 μl was aliquoted into each well of 96-well flat bottom microtiter plates. The clinical isolates of MRSA and *S*. *aureus* ATCC 6538 (control strain) were grown overnight in MH broth were diluted to a 0.5 McFarland turbidity standard in MH broth. A final inoculum of 1.5 X 10^6^ was added to the wells being tested. Bacteria without the addition of antibiotics or PAAG were used as controls. The plates were incubated at 37°C for 24h. The isolates were categorized as sensitive, intermediate, or resistant according to the CLSI guidelines.

### Checkerboard assay

All of the seventeen-drug resistant clinical isolates of methicillin resistant *S*. *aureus* were used for the study, based on their resistance to oxacillin determined by the MIC microdilution assay. The MICs of oxacillin and PAAG in combination were determined using two-dimensional checkerboard microdilution assay with MHB and a final inoculum of 1.5 X 10^6^ CFU/mL. The 96-well plate contained increasing concentrations of oxacillin ranging from 1–128 μg/mL on the x-axis and increasing concentrations of PAAG ranging from 2–256 μg/mL on the y-axis. MICs and fractional inhibitory concentrations (FIC’s) were determined after 24 h growth at 37°C. The FIC of each drug A or B is determined by dividing the MIC of each drug when used in combination by the MIC of each drug when used alone. The FIC_A/B_ of the combination is the sum of each individual FIC: FIC_A/B_ = FIC_A_ + FIC_B_, where FIC_A_ = MIC of A (combination)/ MIC of A alone and FIC_B_ = MIC of B (combination)/ MIC of B alone. The effect of *in vitro* antibiotic combinations is interpreted based on the fractional inhibitory concentration index (FICI) which is described as FICI < 0.5 synergy, 0.5 < FICI < 4 additive effects or indifference, and FICI ≥ 4 antagonism [44–47].

### Time-kill analysis

Seventeen 24-hour experiments including a growth control were used to study the bactericidal effect of PAAG on each of the clinical isolates of MRSA. Bacteria were grown for 24 h at 37°C in MHB. The bacterial culture was further diluted to 0.5 by McFarland standard turbidity. The diluted bacterial culture was serially diluted, and spot plated onto LB agar plates. The initial inoculum was found to be 1.5 X 10^6^ CFU/mL. The bacterial inoculums were treated with PAAG at a concentration of 100 μg/mL and incubated at 37°C for 1, 4, 12 and 24 h. Culture aliquots were removed at 0 h, 1 h, 4 h, 12 h, and 24 h and then serially diluted in sterile water, plated in duplicates onto LB agar plates and incubated at 37°C for 24 h. Cell viability was determined by enumerating the number of visible colonies in the LB plates. Effective bactericidal activity was defined as a 3-log reduction in the CFU/mL from the baseline. Time-kill curves were generated using GraphPad Prism 6 software, by plotting the mean colony counts (log_10_ CFU/mL) versus time.

### PI permeability study

The effect of the PAAG on MRSA permeability was studied by measuring propidium iodide (PI) uptake by bacterial cells. Five MRSA strains (SA10, 2- 4C, 2- 1A, 2-9A and 2- 5A) used in the study demonstrated high resistance to mupirocin and oxacillin (Table 1). Overnight cultures were centrifuged at 13,000 rpm for 1 min. The supernatant was discarded. Bacterial pellets were resuspended in water and diluted to obtain an inoculum of 1 × 10^8^ cells/mL. All assays were performed at room temperature. For each assay, 3 ml of bacterial suspension was mixed with PI (2.5 mg/mL in water) to make a final concentration of 17μg/mL [36]. The mixture was added to the wells of a 96 well plate and fluorescence was measured via SpectraMax Gemini XPS (Molecular Devices). PAAG (50–200 μg/mL), vancomycin, oxacillin, mupirocin, or bacitracin at concentrations of 100 μg/mL or no antibiotic control was added to the wells containing the mixture. The mixture was mixed thoroughly before fluorescence measurements were taken again. Fluorescence was measured at excitation and emission wavelengths of 535 and 625 nm, respectively. Fluorescence intensity was taken every ten minutes for up to 4 hours.

**Table 1:** *In vitro* minimum inhibitory concentration (MIC) of PAAG, oxacillin and mupirocin against *S*. *aureus* and clinical MRSA isolates.

*S*. *aureus* Strains	MIC (μg/mL)		
	PAAG	Oxacillin	Mupirocin
ATCC 6538*	32	<0.125 (S)	<0.125 (S)
MW-2	32	16 (R)	<0.125 (S)
SA01	32	16 (R)	<0.125 (S)
SA02	32	32 (R)	16 (LR)
SA03	16	16 (R)	<0.125 (S)
SA04	32	8 (R)	<0.125 (S)
SA05	32	4 (R)	>64 (R)
SA06	32	8 (R)	16 (LR)
SA07	32	16 (R)	<0.125 (S)
SA08	32	128 (R)	8 (LR)
SA09	32	16 (R)	<0.125 (S)
SA10	32	64 (R)	<0.125 (S)
SA11	32	16 (R)	<0.125 (S)
SA12	16	64 (R)	<0.125 (S)
2-1A	128	256 (R)	>512 (HR)
2-4C	64	64 (R)	>512 (HR)
2-5A	64	128 (R)	>512 (HR)
2-9A	64	128 (R)	>512 (HR)

Clinical resistance breakpoints according to CLSI: mupirocin > 512 μg/mL (high resistance -HR); 8–64 μg/mL (low resistance-LR); oxacillin ≥ 4 μg/mL (resistant–R); oxacillin < 4μg/mL (sensitive-S).

*MIC bacitracin was found to be 200 μg/mL.

### *In vitro* resistance development study

Serial-passage experiments were completed in 96-well plates as a series of individual MIC experiments with a broad range of PAAG, bacitracin and mupirocin concentrations. *S*. *aureus* 6538 was the parental strain selected because it is susceptible to bacitracin and mupirocin. A single colony of *S*. *aureus* ATCC 6538 was inoculated in MHB and grown for 16–24 hours at 37°C. On day 1, each plate (in duplicate) containing serial dilutions of PAAG, bacitracin or mupirocin was seeded with 1.5 X 10^6^ CFU/mL of exponentially growing *S*. *aureus* ATCC 6538. After overnight growth, the MIC was determined, and the highest drug concentration that allowed growth was diluted 1 in 10^−3^ in MHB. The diluted bacteria were used as an inoculum for the next day’s MIC assay following which, plates were incubated overnight as before, and the process was repeated for 20 days. Aliquots of bacteria from each serial-passage experiment were stored at -80°C in MHB supplemented with 15% glycerol [48, 3].

### Re-sensitization of antimicrobial resistant strains

Additional checkerboard assays were performed on mupirocin and bacitracin resistant *S*. *aureus* 6538 samples collected from the 13th and 20th passages respectively. The synergistic effects of PAAG in combination with the antibiotics (bacitracin and mupirocin) were assessed by the checkerboard assay. Two-fold serial dilutions of bacitracin (100–6400 μg/mL) and mupirocin (4–512 μg/mL) were tested individually in combinations with PAAG ranging from 2–256 μg/mL. The FIC’s were calculated as described above.

## Results

### *In vitro* antimicrobial activity

Seventeen clinical isolates of MRSA were acquired from patients with opportunistic respiratory, skin and blood infections. The antimicrobial activity of PAAG, oxacillin and mupirocin against the MRSA clinical isolates was measured using standard planktonic measurements of minimum inhibitory concentrations (MICs). The measured MICs were used to characterize the multidrug resistance in known MRSA strains with respect to their resistance to oxacillin and mupirocin. A minimum inhibitory concentration (MIC) of ≥ 4 μg/mL for oxacillin defines MRSA [43]. All seventeen clinical isolates of MRSA exhibited significant resistance to oxacillin, the clinical resistance breakpoint of oxacillin being ≥ 4 μg/mL (CLSI). Six strains out of the 17 MRSA clinical isolates tested showed resistance as well to mupirocin (MMRSA), the clinical resistance breakpoint for mupirocin being > 512 μg/mL. PAAG MICs against multidrug-resistant (mupirocin and oxacillin) clinical isolates of *S*. *aureus* ranged between 16–128 μg/mL as shown in Table 1.

### Checkerboard microdilution assay

A standard checkerboard microdilution assay was used to test combination treatments of oxacillin and PAAG against planktonic cells of a wide range of clinical isolates of MRSA characterized in Table 1. The fractional inhibitory concentrations (FIC’s) were calculated from the highest dilution of antibiotic combination permitting no visible growth. The FICI values < 0.5, 1.0, and > 4 were defined as synergistic, additive or indifferent, and antagonistic respectively, according to the previously published methods [46, 47]. Table 2 reports the MICs and fractional inhibitory concentration (FIC_PAAG/OXA_) values calculated for combination of PAAG and oxacillin, for each strain tested. PAAG, when used in combination with oxacillin, demonstrated a strong reduction in MIC of oxacillin (upto 128-fold), decreasing the MIC_OXA_ below the clinical sensitivity break points for all the MRSA strains tested (Table 2). The addition of PAAG at concentrations ≤ 8 μg/mL (2 μg/mL being the lowest concentration of PAAG that exhibited synergy) was sufficient to re-sensitize all of the antibiotic resistant clinical isolates of MRSA to oxacillin.

**Table 2:** *In vitro* activities of PAAG in combination with oxacillin against clinical isolates of multidrug-resistant *S*. *aureus*.

*S*. *aureus* Strains	MIC (μg/mL)		FIC* PAAG/OXA	Relationship
	PAAG [with OXA]	OXA [with PAAG)]		
MW-2	2	1	0.2	Synergistic
SA01	2	2	0.2	Synergistic
SA02	4	8	0.4	Synergistic
SA03	4	4	0.5	Synergistic
SA04	4	2	0.4	Synergistic
SA05	4	1	0.4	Synergistic
SA06	4	1	0.4	Synergistic
SA07	4	1	0.3	Synergistic
SA08	4	2	0.1	Synergistic
SA09	8	4	0.5	Synergistic
SA10	16	1	0.5	Synergistic
SA11	2	4	0.3	Synergistic
SA12	2	1	0.1	Synergistic
2-1A	4	2	0.2	Synergistic
2-4C	8	1	0.2	Synergistic
2-5A	4	1	0.3	Synergistic
2-9A	16	1	0.3	Synergistic

*FIC_A/B_ = FIC_A_ + FIC_B_, where FIC_A_ = MIC of A (combination)/ (MIC of A alone) and FIC_B_ = MIC of B(combination)/ (MIC of B alone). The FICI interpretation used was FICI < 0.5 synergy, 0.5 < FICI < 4 additive effects or indifference, and FICI ≥ 4 antagonism.

### Time-kill assay

The MIC and the checkerboard assays confirmed PAAG’s ability to inhibit bacterial growth of MRSA clinical isolates for the standard 24-hour assay period. The time-kill study further characterized PAAG kinetics of bactericidal activity. PAAG was used at a concentration of 100 μg/mL and compared to untreated controls for all seventeen MRSA strains tested. Samples were plated to determine colony forming units (CFU) in triplicate (n = 3) for the MRSA strains at time points 0, prior to the introduction of PAAG, and at 1, 4, 12, and 24 h after the addition of PAAG.

**Fig 1:**
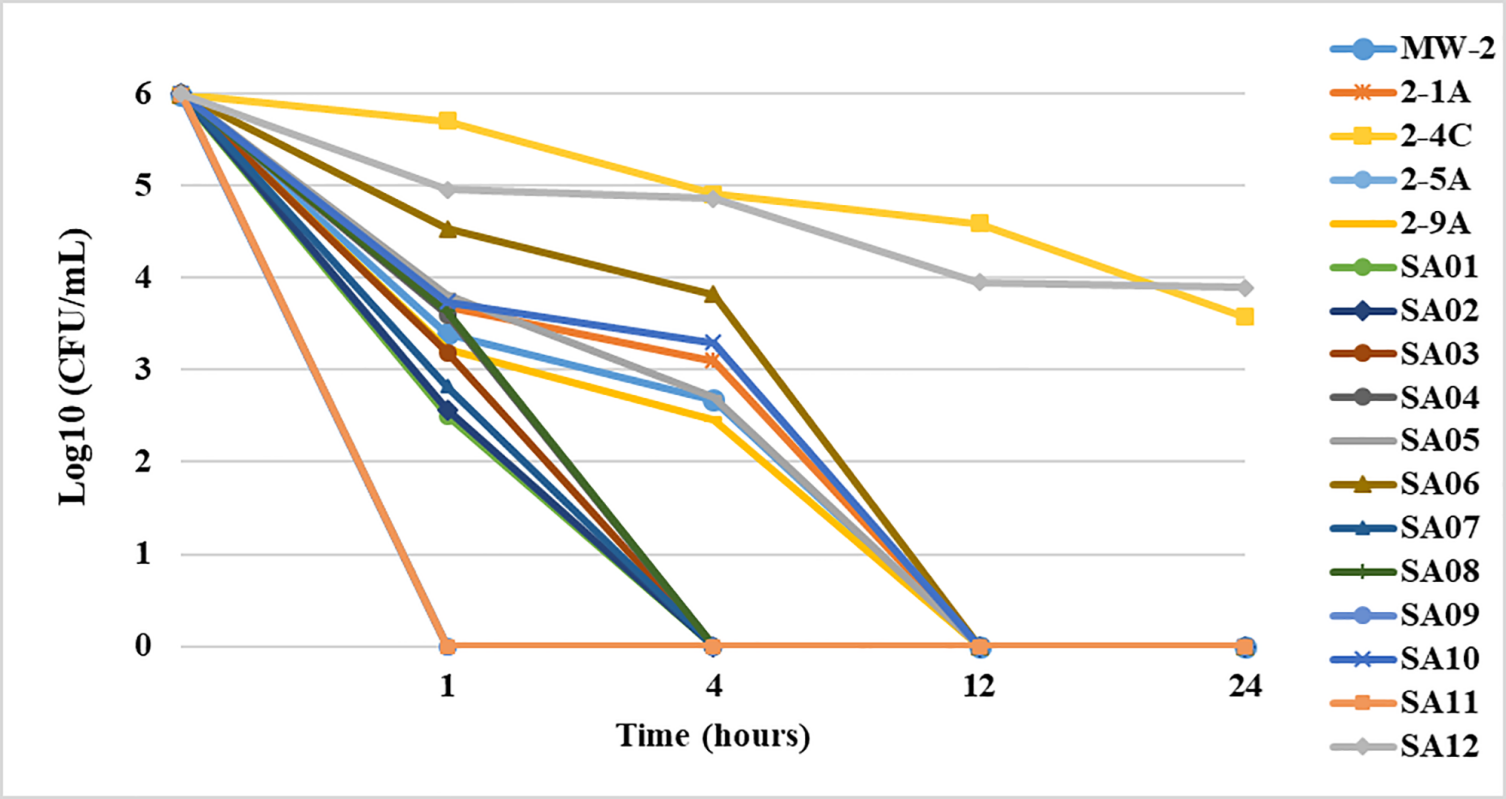
Bactericidal activity of PAAG against seventeen MRSA clinical isolates. PAAG at a concentration of 100 μg/mL was added at timepoint 0 and monitored until 24h. Six log reductions in CFU/mL were observed in 58% of the MRSA isolates tested in 1-4h of treatment. 88% of the MRSA strains was observed within 12h of PAAG treatment. Data is presented as mean ± SD (n = 3).

Fig 1 shows the time-dependent killing of the clinical MRSA strains with the addition of PAAG. PAAG (100 μg/mL) demonstrated rapid bactericidal activity against 58% of the MRSA isolates tested, eliminating a starting inoculum of 1.5 x 10^6^ CFU/mL within 1- 4h. A 2-log reduction in the CFU/mL was observed upon 1 h treatment of PAAG for 15 of the 17 clinical isolates tested. PAAG completely cleared 88% of the MRSA isolates with 6- log reduction in CFU/mL upon 12 h of treatment with PAAG. PAAG exhibited slower bactericidal activity against strains SA10 and 2-4C with < 2 log reduction (CFU/mL) in 24 hours. Complete elimination of these two strains was not possible even after 24 hours at PAAG concentrations of 100 μg/mL.

### Permeabilization of MRSA by PI uptake assay

**Fig 2:**
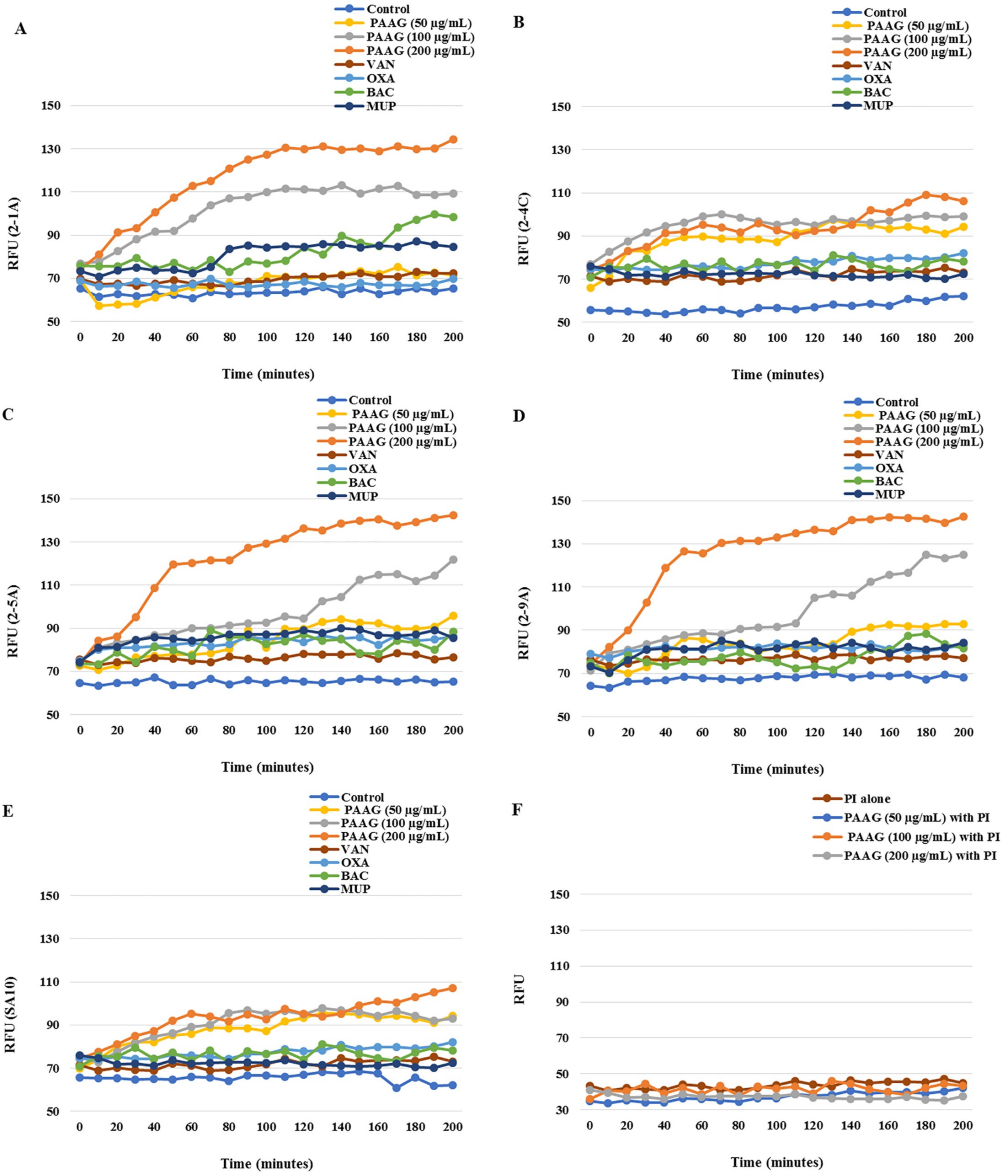
**(A-F).** The relative fluorescence units (RFU) measured reflects PI intercalation into bacterial DNA by 2-1A (A), 2-4C (B), 2-5A (C), 2-9A (D), SA10 (E), PI alone and PI with PAAG (F) over 200 minutes, with the addition of control, 50–200 μg/mL PAAG, 100 μg/mL of vancomycin, oxacillin, mupirocin or bacitracin respectively.

To assess the mechanism of antibacterial activity of PAAG, the PI uptake assay was used to measure bacterial permeabilization by PAAG. PI shifts emission upon intercalation into the bacterial DNA. Untreated cells did not display any PI fluorescence intensity. (Fig 2A–2E) shows the relative fluorescence unit (RFU) of emission showing PI fluorescence on exposure of 50–200 μg/mL of PAAG and 100μg/mL of oxacillin, bacitracin, mupirocin and control. A steady increase in fluorescence intensity was observed with increasing PAAG concentrations for isolates 2-1A, 2-5A and 2-9A. The fluorescence intensity increased dramatically within 30 minutes of addition of PAAG (200 μg/mL) when compared to the antibiotics (Fig 2). The increase in fluorescence in treated samples clearly indicates the permeabilization of bacterial cells as a result of PAAG treatment.

Strains SA10 and 2-4C were observed in the time kill assay to be less affected by PAAG treatment. Treatment with PAAG resulted in minimal increase in PI fluorescence intensity for strains SA10 and 2-4C compared to the other strains. The lack of significant increase in intensity upon PAAG treatment on these two strains explains the slower bactericidal action of PAAG observed in the time kill curves. These strains will be further investigated in future studies.

Vancomycin, oxacillin, bacitracin and mupirocin showed minimal increase in fluorescent intensity even after 4h of treatment, as shown in Fig 2. While bacitracin, oxacillin and vancomycin are known to disrupt cell wall synthesis, mupirocin reversibly inhibits bacterial protein and RNA synthesis. None of these traditional antibiotics act upon contact to permeabilize bacteria, and thus the potential bactericidal effects may take longer than the duration of this study. Control experiments were carried out by adding PI onto the bacteria, in the absence of PAAG. No change was observed for the fluorescence intensity of PI alone and PI with PAAG (Fig 2F).

### Antimicrobial resistance study

**Fig 3:**
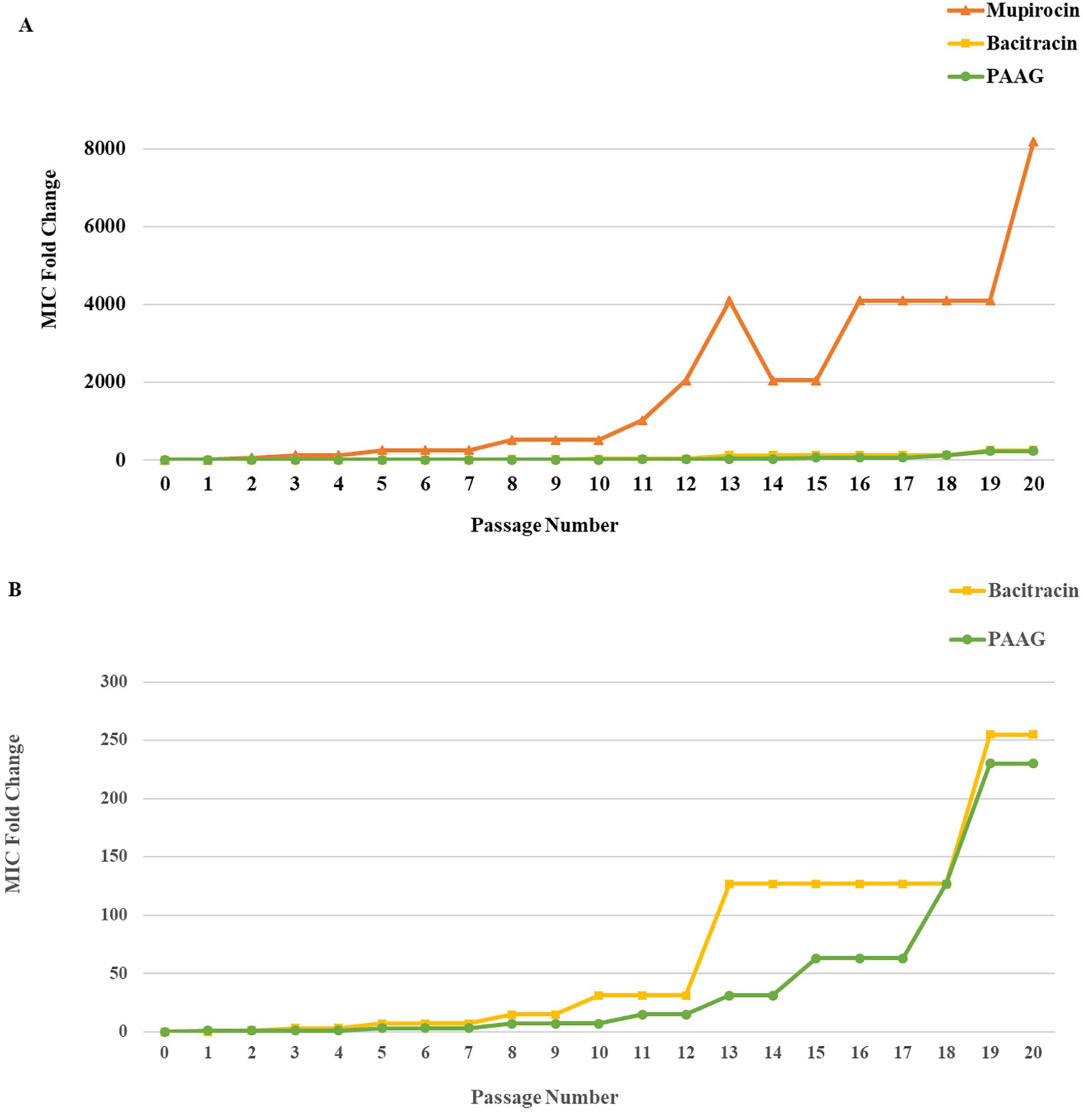
*In vitro* resistance development study. Development of *in vitro* resistance to PAAG was compared to that of two other topical antibiotics, mupirocin and bacitracin, plotted as MIC fold change vs passage number (A). *S*. *aureus* ATCC# 6538 developed resistance to PAAG and bacitracin at a slower rate compared to the rapid onset of resistance to mupirocin. Fig 3B is an expansion of Fig 3A showing the MIC fold change to PAAG and Bacitracin.

The results of the serial incubation experiments are presented in Fig 3 as the fold changes in MIC plotted against number of incubations. All experiments demonstrated emergence of resistance, allowing *S*. *aureus* to grow in successively higher concentrations of mupirocin, bacitracin and PAAG over time. After 20 days of serial passage, the MICs were found to be 1024 μg/mL for mupirocin (MIC > 512 μg/mL classified as high resistance (CLSI)), 51 mg/mL for bacitracin, and 8194 μg/mL for PAAG. The general trend toward bacterial growth in higher concentrations of PAAG and bacitracin resulted from a series of small incremental changes in MIC over the course of 20 days, as opposed to mupirocin, where an early dramatic change in MIC was followed by similar increases upon subsequent passages.

### Resensitization of the antimicrobial resistant strains

Additional checkerboard assays were performed on mupirocin and bacitracin resistant strains of *Staphylococcus aureus* 6538 collected at 13th and 20th serial MIC passage. PAAG in combination with bacitracin re-sensitized the bacitracin resistant *Staphylococcus aureus* ATCC# 6538 displaying a reduction up to 256 -fold in the MIC. PAAG concentrations as low as 4 μg/mL, significantly reduced MICs of bacitracin from both 25.6 mg/mL and 51.2 mg/mL to 0.2 μg/mL for both passage 13 and 20, respectively. PAAG (4 μg/mL) also re-sensitized highly mupirocin-resistant isolates of *S*. *aureus* 6538, facilitating substantial reduction in MICs from 1024 μg/mL (passage 20) and 512 μg/mL (passage 13) to 0.125 μg/mL, resulting in a 4 order of magnitude change in MIC. MICs of mupirocin and bacitracin for these resistant isolates of *S*. *aureus* were reduced below their clinical susceptibility breakpoints (≤ 2 μg/mL) [43]. The assay demonstrated the ability of PAAG to potentiate the activity of antibiotics in a strain with recent resistance development, there by re-sensitizing *S*. *aureus* to bacitracin and mupirocin.

## Discussion

The panel of isolates tested represent a collection of a wide range of clinical isolates, associated with respiratory, skin and blood infections. All the isolates tested were found to be resistant to oxacillin and 35% of the clinical isolates of MRSA tested were found to be highly resistant to mupirocin (Table 1), according to the CLSI standards [43]. PAAG demonstrated potent antibacterial activity against all the multidrug resistant clinical isolates of MRSA tested, with MICs ranging from 16–128 μg/mL (Table 1). The antimicrobial activity exhibited by PAAG is hypothesized to be a result of its polycationic nature, and is a result of permeabilization of the bacterial membrane, as shown in Fig 2 and demonstrated previously for its interaction with the Gram-negative *E*. *coli* [36].

Oxacillin has been used in combination with vancomycin in clinical use, though has not been proven to be a therapeutically effective combination in treating MRSA infections. PAAG when combined with oxacillin exhibited strong synergy against all seventeen clinical isolates of MRSA tested with FIC ranging from 0.1 to 0.5. The MIC for oxacillin was reduced up to 128-fold in the presence of PAAG. No antagonistic interactions were observed between PAAG and oxacillin for all strains tested. PAAG was able to decrease the MIC of oxacillin below clinical susceptibility breakpoints of <4 μg/mL for 94% of the MRSA strains tested (16/17). The direct antimicrobial and synergistic activities of PAAG suggest its potential to be used alone or in combination with oxacillin to treat infections that would otherwise be resistant to oxacillin. These findings are supported by similar observations for PAAG in potentiating the activity of antibiotics against multidrug-resistant *Burkholderia* isolates [37]. The fact that synergistic, and not additive, effects were observed is hypothesized to reflect that these drug resistant *S*. *aureus* strains are likely less fit than sensitive strains. Multidrug resistance in bacteria is often linked to a fitness cost associated with utilizing a less efficient system or the energy cost of antibiotic pumps and clearance efforts, which explains their reduced growth rates and virulence [49, 50]. If a strain is less fit, the ability to respond to a pore-forming agent could be less robust, resulting in a synergistic rather than additive effect of the combination therapy.

PAAG’s ability to eliminate bacteria through direct bactericidal activity, in addition to inhibition of bacterial growth, was assessed though time-kill assays. The time dependent reduction in CFU/mL of all the 17 clinical isolates over a 24-hour period post treatment with PAAG (100 μg/mL) has been shown in Fig 1. Treatment with PAAG at a concentration of 100 μg/mL resulted in eradication of 88% (15/17) of the multidrug resistant strains followed by a 6-log reduction in CFU/mL. Strains highly resistant to mupirocin (MIC >512 μg/mL) were eradicated (> 5 log reduction) within 4 hours of treatment with PAAG (100 μg/mL). Most commonly isolated opportunistic respiratory pathogens such as MW-2 and 2-1A was found to be eradicated (6- log reduction) with in 1 hour of treatment with PAAG (100 μg/mL). It was noted that PAAG at concentrations 3x MIC (100 μg/mL) exhibited slower bactericidal activity against SA10 and 2-4C with < 2 log reduction in CFU/mL in 24 hours. In the clinical setting, bacteria exposed to sublethal doses of antibiotics may accumulate point mutations resulting in amino acid substitutions that result in increased MICs. Specifically, mutations in mprF, yycG, and rpoB have demonstrated treatment associated increases in MICs and suggest that genetic changes in these genes can influence antimicrobial susceptibility [51]. Studies have also shown that bacteria can resist killing by peptides, such as AMP’s, by membrane modifications, active efflux and reduced uptake [52]. These resistant strains might have a phenotype defined by increased cell wall thickness and increased membrane fluidity [53]. Therefore, it is hypothesized that these altered membrane arrangements may be limiting efficient PAAG insertion into the membrane [54–56]. Slower bactericidal activity of PAAG against SA10 and 2-4C was further analyzed and investigated using a PI uptake study.

Bacterial permeabilization upon treatment with PAAG and standard antibiotics were assessed using a fluorescent probe, propidium iodide (PI). PI cannot enter the bacteria unless the outer layer of the cell is permeabilized. If PI enters the bacteria, DNA-bound PI fluoresces with excitation and emission at 544 nm and 620 nm, respectively. The results of the study confirm that bacteria treated with PAAG are leaky, allowing increased uptake of PI. PAAG rapidly permeabilized five of the MRSA isolates tested in a dose dependent manner as indicated by an increase in PI fluorescence (Fig 2). The antibiotics tested showed minimal increase in PI intensity, even after 4h of treatment, which helps to explains bacterial persistence. One of the major reasons for emergence of antibiotic resistance is poor permeability of the outer cell wall to antimicrobial agents [57]. Vancomycin resistance arises from thickening of the cell wall followed by the segregation of vancomycin at non-active cell wall targets [3, 57]. Permeabilization of the outer cell wall and membrane might play a role in the mechanism of PAAG mediated killing of the clinical MRSA isolates and is likely a contributor to PAAG’s synergistic activity. Similar to antimicrobial peptides (AMP’s), polycationic PAAG most likely first interacts with the net negative charge of the bacterial cell surface, followed by disruption of membrane integrity.

Treatment with PAAG did not lead to as much PI uptake in strains SA10 and 2-4C compared to the other three strains. A number of known cell wall and membrane resistance mechanisms [54–56] could account for the reduced permeabilization of these two strains. Further studies on the changes in cell wall and membrane composition of these resistant strains will be conducted to better understand the slower killing exhibited by PAAG on SA10 and 2-4C.

The investigation of the development of resistance of *S*. *aureus* 6538 to PAAG, mupirocin and bacitracin is shown in Fig 3, where the fold change in MIC is shown over the course of 20 passages. Mupirocin and bacitracin were selected as comparators as they have very different mechanisms of action. Bacitracin disrupts Gram-positive bacteria by interfering with cell wall synthesis and is used topically. Mupirocin selectively binds to bacterial isoleucyl-tRNA synthetase, which stops or slows bacterial protein synthesis. Also, mupirocin is known to have genetic transfer of resistance [58, 59] and bacitracin is known to develop resistance slowly, compared to other conventional antibiotics [60].

Mupirocin was observed to express nearly a 63-fold increase in MIC following the first passage, suggesting that the genes for the resistance were available. A rapid increase in resistance continued, leading to >8000-fold increase in MIC, over the next 20 passages. Conversely, both PAAG and bacitracin exhibited slower development of resistance, with incremental changes upon passages up to slightly greater than 200-fold increase in MIC by 20 passages. While studies are ongoing to unravel the mechanism of PAAG resistance, these results shed light to the point that both PAAG and bacitracin interactions with the cell walls result in similar time lines for developing resistance in this ATCC strain. However, this observation may not be generalizable to all strains.

PAAG displayed substantial potential to re-sensitize mupirocin and bacitracin resistant *S*. *aureus* isolated from passages 13 and 20 below clinical sensitivity breakpoints (≤ 2 μg/mL) [43]. Synergistic combinations of PAAG at concentrations as low as 4 μg/mL with mupirocin/ bacitracin resulted in a 4-order magnitude reduction in the MICs of mupirocin and bacitracin respectively.

Combination therapies have been reported to minimize the likelihood of resistance development, to reduce drug toxicity by lowering the efficacious dose and to broaden the range of pathogenic bacteria that can be targeted [3, 61]. While the MICs of antibiotics are used to quantify the *in vitro* antimicrobial activities of antibiotics against infectious microorganisms, consideration of bactericidal effects of the drug is also a key factor to consider when determining antibiotic dosing regimens. The findings of the current study point to the potential of PAAG as a means to treat MRSA infections through a direct bactericidal activity or through combination therapies. PAAG was observed to enhance the antibacterial activity of oxacillin, potentially increasing its clinical efficacy. PAAG was also shown to re-sensitize a resistant strain of *S*. *aureus* to both mupirocin and bacitracin, suggesting that PAAG could potentially expand the range of multidrug-resistant bacteria that can be treated, should these observations continue for a broader range of bacteria [4].
